# Integrated laboratory workflow for proton exchange membrane fuel cell fabrication and testing

**DOI:** 10.1038/s41598-025-27923-9

**Published:** 2025-12-01

**Authors:** Haya Hesham, Mohamed Abdel Rahman, Ghada Bassioni, Rania A. Swief, Mohamed Ezzat, Sherif Helmy, Nourhan M. Elbehairy

**Affiliations:** 1 Electrical Power and Machines Department, Faculty of Engineering, Ain Shams University, Cairo, Egypt; 2 Engineering Physics and Mathematics Department, Faculty of Engineering, Ain Shams University, Cairo, Egypt; 3 Electrical Power and Energy Department, Military Technical College, Cairo, Egypt; 4 Energy and Renewable Energy Department, Faculty of Engineering and Technology, Egyptian Chinese University, Cairo, Egypt

**Keywords:** PEMFC, GDL, CCM, MEA, Fabrication, Assembly, Temperature, RH, Flow rate., Energy science and technology, Engineering, Materials science

## Abstract

This paper is a laboratory experiment aimed at fabricating the different layers that make up the Proton Exchange Membrane Fuel Cell (PEMFC), assembling it, constructing the cell, and then testing it under different operating conditions of temperature, relative humidity, type of reactant gases (air vs. oxygen), and flow rate of reactant gases into the cell. At the fabrication phase, the materials used, their quantities, as well as the detailed steps required to fabricate each layer are clarified in detail. In the testing and evaluation phase, the fabricated cell is connected to a test station, and all operating conditions are controlled; then voltage, current, and output power are measured. Through this experiment, it can be concluded that the best performance of PEMFC can be obtained when reaching the required loading of the coating materials on the different layers, as well as when distributed regularly on the substrate. As for the operating conditions, there is an improvement in the cell performance when raising temperatures and when it is operated at values of relative humidity between 80% and 100% and when oxygen flows instead of air as an oxidant in the reaction which occurs inside the cell.

## Introduction

The world has recently witnessed a significant increase in the percentage of electricity generated from renewable energy sources, as the latest statistics announced by the US Energy Information Administration (EIA) state that the global electricity capacity reached 9,080 GW in 2023, of which 3,833 GW are from renewable energy sources such as solar, wind and hydropower. These statistics also show that the total global electricity generation in the same year reached 29,124 TWh, of which 9,004 TWh are from renewable energy sources^1^. Due to the interest of all countries in increasing the contribution of renewable energy sources and the diversity of their types, interest in Hydrogen energy and Proton Exchange Membrane Fuel Cells (PEMFCs) has emerged as one of the technologies used in this field. This cell converts chemical energy into electrical energy through the chemical reaction between hydrogen and oxygen. It is a clean electrochemical energy conversion device that produces only water and heat without air pollutants. Transportation and portable power applications are common applications in which this type of fuel cell is used due to its compact size and high power density^2–4^. One of the features of this cell is the low operating temperature, as it operates at temperatures ranging between 60 °C and 100 °C, which leads to rapid start-up, although this leads to an increase in the sensitivity of the catalyst layer in the cell to the impurities in the operating fuel, such as carbon monoxide (CO), as it may lead to poisoning the platinum-based catalyst^2,5^. The life span of this cell may vary according to the type of application used, reaching 30,000 h in the case of stationary applications and up to 2500–3000 h in the case of automotive applications^6^. In this study, the fabrication of different layers of the cell and their assembly in the laboratory will be examined and then the performance of the cell under different conditions will be evaluated.

One of the main components of PEMFCs is the Membrane Electrode Assembly (MEA), which is a proton-conducting membrane that is covered by catalyst layers – Catalyst Coated Membrane (CCM), followed by gas diffusion layers (GDLs). It determines the performance and durability of the cell^7^. The function of MEA is to efficiently transport the protons from one electrode to another, distribute the gases within the cell, and regulate water management. It must be fabricated and assembled appropriately for optimal performance under different operating conditions^8,9^. The fabrication process of MEA has witnessed great development and new technologies recently^10,11^. Among the most famous fabrication methods are hot pressing, spray coating, and electrospinning, as it ensures uniform distribution of materials on the substrate and strong adhesion to them and reduces internal resistance^2^. Research has also focused on the cost of catalyst fabrication while maintaining high cell performance by using lower-cost materials in fabrication such as Sulfonated Poly (Ether Ether Ketone) (SPEEK) instead of Nafion^12^. GDL is an essential layer in MEA as it helps to diffuse reactant gases and remove water resulting from the reaction while maintaining the mechanical stability of the cell. PUREBLACK carbon is used to fabricate GDL in reference^13^; its first experimental use was in lithium-ion batteries^14^. The use of PUREBLACK carbon improves the performance of the cell when operating under different levels of humidity as it is characterized by a partly graphitized core shell, and its porosities are uniform and graded^15,16^. The development of MEA fabrication is discussed, and different methods and technologies are reviewed in^17^ to achieve the highest efficiency and longest cell life span. The design and material of the flow fields in the plates that represent electrodes have an active role in the performance of the cell^18,19^. The study in reference^20^ proves that cell performance improves when using Optimized Multi-Parallel Serpentine Flow Fields instead of Conventional Flow Fields, as it leads to better water management and more uniform current density distribution. The experimental study in reference^21^ is concerned with the effect of operating conditions on cell performance and reviews the polarization curves of a 30-cell stack which relies on cells with a membrane of Nafion-212.

Recent studies have focused on catalyst improvement, reducing the loading of the materials used in MEA fabrication, flow field design optimization, or membrane conductivity enhancement, focusing on a single variable change. However, most of these studies depend on commercial components existing in the market or modelling and simulation of the tested cell.

In this study, the different layers that make up the MEA will be fabricated in the laboratory, then these layers will be assembled to construct the cell and then the cell performance will be tested under different operating conditions.

During the fabrication phase, the effect of changing the materials used, changing their quantity, and the method of distributing them on their substrate on the performance of the cell will be studied. During the assembly phase, the effect of adding a layer of carbon paper on both sides of the MEA and how this layer affects cell performance will be studied. During the cell testing phase, the cell will be exposed to different operating conditions such as temperature, humidity, type of gases and their flow rate, and the effect of this change on cell performance and output power, voltage, and current values will be studied by plotting a polarization curve to get the best operating conditions that give the optimal cell performance.

This study contributes a methodological and integrative advance. The novelty is the detailed end-to-end reproducible in-lab fabrication, assembly, activation and testing protocol of the cell. Unlike most previous studies, this study presents a detailed workflow and investigates the effect of MEA structure, material type, material processing, coating distribution and loading, simple quality control checks, assembly execution and systematically varying operating conditions on cell performance, all of these factors combined together, to achieve the optimal performance practically. It is proved throughout the study that the implementation process can be considered as an important design variable rather than a secondary consideration.

The integration of the activation protocol of the used membrane material as a material processing enriches the analysis of cell performance. Detailed activation procedure of the fuel cell that includes break-in procedure and air starvation technique, considering the duration and electrical current setpoints, makes the study more integrated.

Furthermore, it is quantified how changes in operating variables affect peak power gains, for example, the percentage increase resulting from a 10 °C temperature step or from using oxygen instead of air. In addition, the pre-collapse current, which is the maximum current the cell can produce immediately before voltage collapse, is tracked as an integrative stability limit beyond peak power. Accordingly, cell performance is studied in a different manner, which takes into consideration using the cell as a power supply feeding an electrical load, from previous studies.

The structure of this paper is as follows: Section I is an introduction to the PEMFC. Section II covers the fabrication and Assembly of PEMFC. It also covers the experimental setup. Section III discusses the results of testing the fabricated fuel cells under different conditions. Lastly, Section IV represents the conclusion of the work.

## Methodology and experimental setup

This section will cover the following:

1. MEA Fabrication: The process of GDL and CCM fabrication in the lab will be explained.
2. Fuel Cell Assembly: Assemble the fabricated GDL and CCM to prepare the MEA and make a single cell.
3. Operating Conditions: The cell will be tested at different operating conditions of temperature and humidity under controlled gas flow rate and back pressure values.
4. Performance Evaluation: The performance of the cell will be assessed using Polarization Curves (V-I Characteristics).

### Gas diffusion layer (GDL) fabrication

GDL is the layer that exists between the bipolar plate and the catalyst layer. It is a sub-component of the MEA. Its function is to distribute the entered reactant gases before the reaction to the catalyst layer and to help remove the produced water after the reaction effectively without losing the electrolyte moisture^22^.

Its substrate is a 10*10 $\:{cm}^{2}$ carbon paper (non-woven, teflonized carbon paper (GD07508G) from Hollingsworth & Vose Company, USA). The required carbon loading is 3 mg/$\:{cm}^{2}$. So, the paper needs 300 mg of carbon loading, but about 50% is lost while spraying the ink on the paper, so 600 mg is used. Carbon loading must have 15% vapor-grown carbon nanofiber (VGCF), which is a reinforcement agent, and 85% superior graphite, which is nanochain (core-shell). So, 600 mg carbon will have 90 mg VGCF and 510 mg superior graphite. The required mixture is 70% carbon and 30% PTFE (Teflon). So, 600 mg carbon needs 260 mg PTFE. PTFE in the lab is a 60 wt%, 1.51 g/mL density dispersion^23^, meaning that every 1 g of the dispersion contains 600 mg of PTFE. So, 0.43 g or 0.287 mL is the required amount of the dispersion to get 260 mg of the PTFE. As mentioned in^13^, 0.5 g of carbon needs 120 mg of sodium dodecyl sulfate (SDS). This experiment uses 600 mg of carbon, so it needs 144 mg of SDS. Finally, 20 mL of deionized water is used for the mixture.

After preparing the ink, it will be sprayed using a gun to be distributed equally on the carbon paper. The flow must be slow and consistent. The ink should be sprayed 4–5 times in intervals with breaks of 2 min in between for the carbon paper to dry.

Then, the carbon paper will be dried overnight in an oven at less than 80 $\rm ^\circ C$ to avoid cracks on the GDL surface. The next step is to be sintered for half an hour at 350 $\rm ^\circ C$. The sintering temperature and duration are selected based on the manufacturer’s published product specifications, which indicate a typical particle diameter of 0.2 μm and the following thermal behavior: the dispersion employs non-ionic surfactants; water is removed at 120 °C, wetting agents are removed at 270 °C, and the PTFE melts above 337 °C^23^. The sintering setpoint is selected at 10$\:\to\:$20 $\rm ^\circ C$ above the melting temperature^24–27^.

As a pre-assembly quality-control (QC) check, the final GDL product can be immersed in deionized water overnight as a test to ensure that it gets moist so it can let the gases and water flow through it when put in the fuel cell to ensure good performance. Then, it is set in the oven to dry at 65 $\rm ^\circ C$ for minutes to be used in the fuel cell. The purpose of this process is to verify that all ink components are sound and that the sprayed ink adheres properly to the carbon paper. After deionized water immersion, any ink shedding, flaking, or delamination indicates a spraying process defect or a faulty ink component. By contrast, GDLs that remain intact and show no visible particle release are considered acceptable for cell assembly.

To make sure that the GDL is hydrophobic enough for good fuel cell performance, the used carbon paper for making it can be teflonized before spraying the ink. The steps for carbon paper teflonization are as follows:

- Add 10 mL of the Teflon dispersion (PTFE) to 100 mL of deionized water.
- Put the whole carbon paper in the solution for 5 min. This step is called dip coating.
- Get the paper out of the solution and let it dry without touching any surface.
- Repeat the previous two steps for 2 or 3 times.
- Put the paper in the oven overnight at 80 $\rm ^\circ C$.
- Then, put it in the oven for 30 min at 350 $\rm ^\circ C$.
- Spray the paper with the ink, but SDS does not need to be added now.

Although contact angle measurements have not been made to ensure sufficient hydrophobicity of the GDL, hydrophobic adequacy is assessed indirectly through operational indicators such as no ink shedding/flaking/delamination after GDL immersion in deionized water, open circuit voltage (OCV) stability, no voltage oscillations which is an indication for cathode flooding and that polarization curve can be reproduced with minimum changes under the same operational conditions.

The carbon paper must be weighed before and after spraying the ink to ensure the GDL carbon loading. For example, the weight of a 10*10 $\:{cm}^{2}$ carbon paper before spraying the ink is 2.058 g and its weight after spraying the ink and heating is 2.338 g. So, the carbon weight on the paper is 0.28 g or 280 mg. The carbon loading is 280 mg on 100 cm^2^ paper so it is 2.8 mg/$\:{cm}^{2}$.

### Catalyst coated membrane (CCM) fabrication

The coating layer on the membrane is the catalyst layer, so it is the layer that exists between the GDL and the membrane in the MEA. It facilitates the electrochemical reactions that convert hydrogen and oxygen into electricity and water. The CCM is fabricated according to the following steps:

- Add 100 mg of platinum (TEC10E50E, 47.1% Pt on carbon, Tanaka – Pt/C).
- Add a minimum amount of deionized water to moisten the platinum to avoid sparks when adding the alcohol.
- Add 2 mL of IPA (isopropyl alcohol).
- Add 0.12 mL of Nafion (LIQUION LQ-1105, 5 wt % Ion Power, Inc.) dispersion.
- Stir the mixture slowly overnight.
- Spray one side of the Nafion sheet (Membrane NR-211 of thickness 25.2 μm and weight: 50 g/m², Ion Power) using the gun. The sprayed area = 5 cm^2^.
- Put it in the oven for less than 15 min to dry at 50 $\rm ^\circ C$.
- Spray the other side of the Nafion sheet, then put it once again in the oven for less than 15 min to dry at 50 $\rm ^\circ C$.

The same platinum loading is used for both sides of the Nafion sheet, the cathode and the anode. Although higher cathode loading is common because oxygen reduction reaction is slower than hydrogen oxidation rection, the goal is to eliminate catalyst-mass distribution as a confounder while mapping fabrication and operating-condition effects. Asymmetric allocation introduces microstructural differences (porosity, ionomer content, thickness) between sides, complicating attribution.

The required platinum loading is 0.125 mg/$\:{cm}^{2}$. So, this amount of the prepared ink is sufficient to make 20 catalysts with an area of 5 $\:{cm}^{2}$ taking into consideration that 50% of the ink is lost during the ink spraying process. So, 100 mg of TEC10E50E, 47.1% Pt on carbon, Tanaka – Pt/C contain 47.1 mg of platinum, 50% is lost which means 23.55 mg of platinum. Each catalyst is covered by 1.1775 mg on both sides (0.5888 mg on each side) and its area is 5 $\:{cm}^{2}$ so 0.118 mg/$\:{cm}^{2}$ is the actual loading and accepted.

The Nafion sheet can be replaced by the crosslinked sulfonated polybenzimidazole (PBI) membrane, but it must be activated first as follows:

- Soak the membrane either in 2 M sulfuric acid solution at room temperature for 24 h or in 1 M sulfuric acid solution at 80 °C for 10 h.
- Wash the membrane thoroughly with deionized water until it reaches neutral pH, which is 7; this can be done using pH strips.
- Dry the membrane in an oven at 120 °C overnight to prepare it for the fabrication process.

### Membrane electrode assembly (MEA) assembly and fuel cell construction

**Fig. 1:**
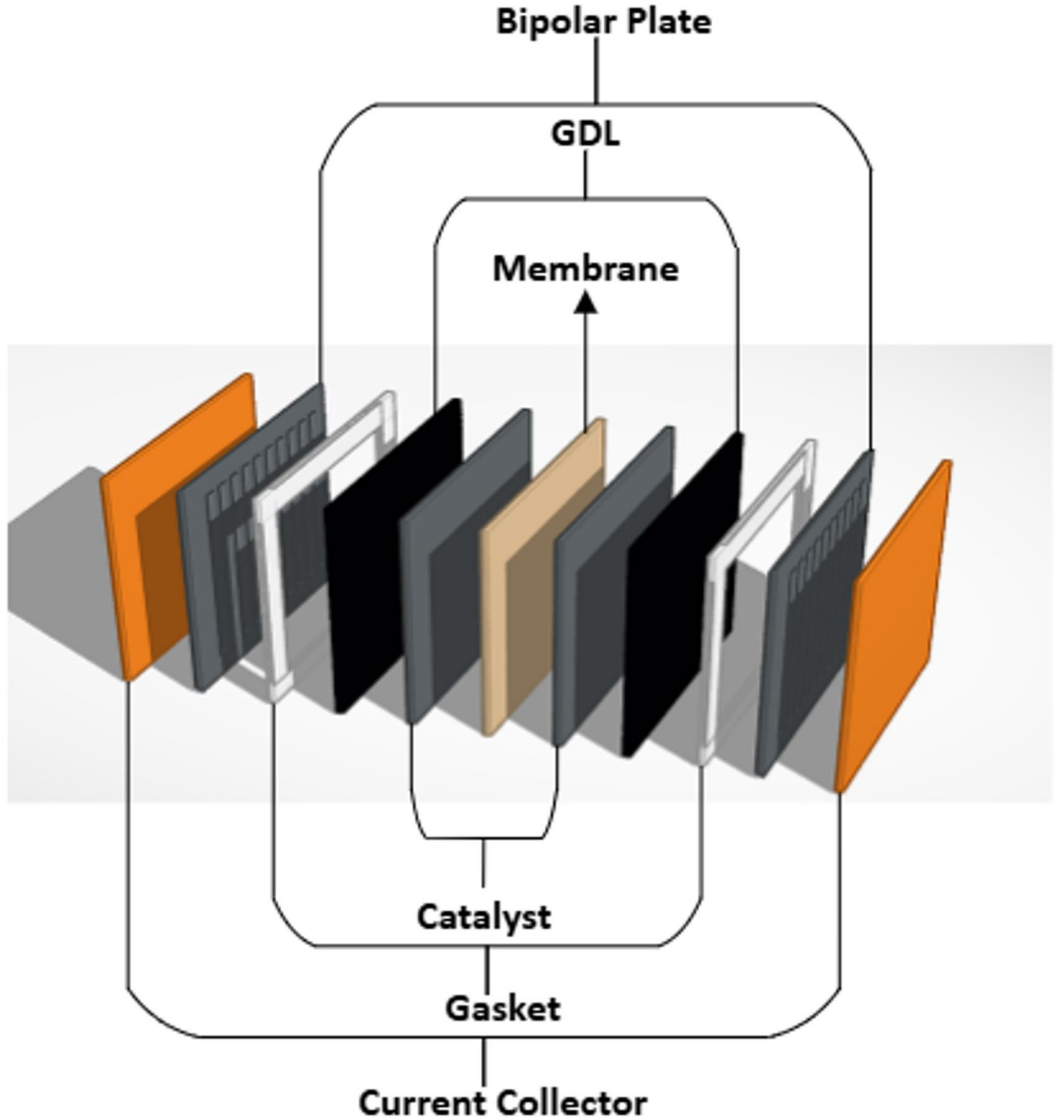
PEMFC layers in detail.

A specific process of assembling the PEMFC is illustrated through the following steps to ensure optimal cell performance. Figure 1 shows the different layers used in the fuel cell assembly.

- Start with the CCM, put a layer of GDL on its two faces ensuring that the sprayed face of the GDL is in direct contact with the CCM. That happens after cutting the fabricated GDL to match the exact dimensions of the CCM to avoid any misalignment.
- Put the gasket layer after the GDL. It is a layer that prevents the gases from leakage during the cell operation. Make sure that it is clean and free of any carbon residues to ensure that it performs its function appropriately and that it does not touch the GDL layer but only surrounding it.
- Use bipolar plates with a conventional single-serpentine configuration of flow fields, which facilitate the flow of Hydrogen, Oxygen, and water in and out of the cell. Put these plates, which are the anode and cathode of the cell, on both sides of the MEA.
- Put a current collector on both sides of the cell that has been built. The current collector is made of copper, allowing the electric current generated by the cell to pass through with the minimum resistance.
- An insulation plate and an end plate are then put on both sides of the cell.
- The cell is then secured by 8 hex-head bolts controlled by 40 LBS torque chosen based on the cell area of 5 cm²^28–30^ so that the pressure inside the cell is distributed optimally for the best cell performance while preventing GDL damage due to excessive compression or gas leakage due to low pressure.
- The fuel cell can now be connected to the Greenlight Test Station (G40, Hydrogenic, Canada). This test station can control the operation conditions such as temperature, humidity levels, gas flow rates, and back pressure and measure the output power, current, and voltage so the cell performance can be assessed.

**Fig. 2:**
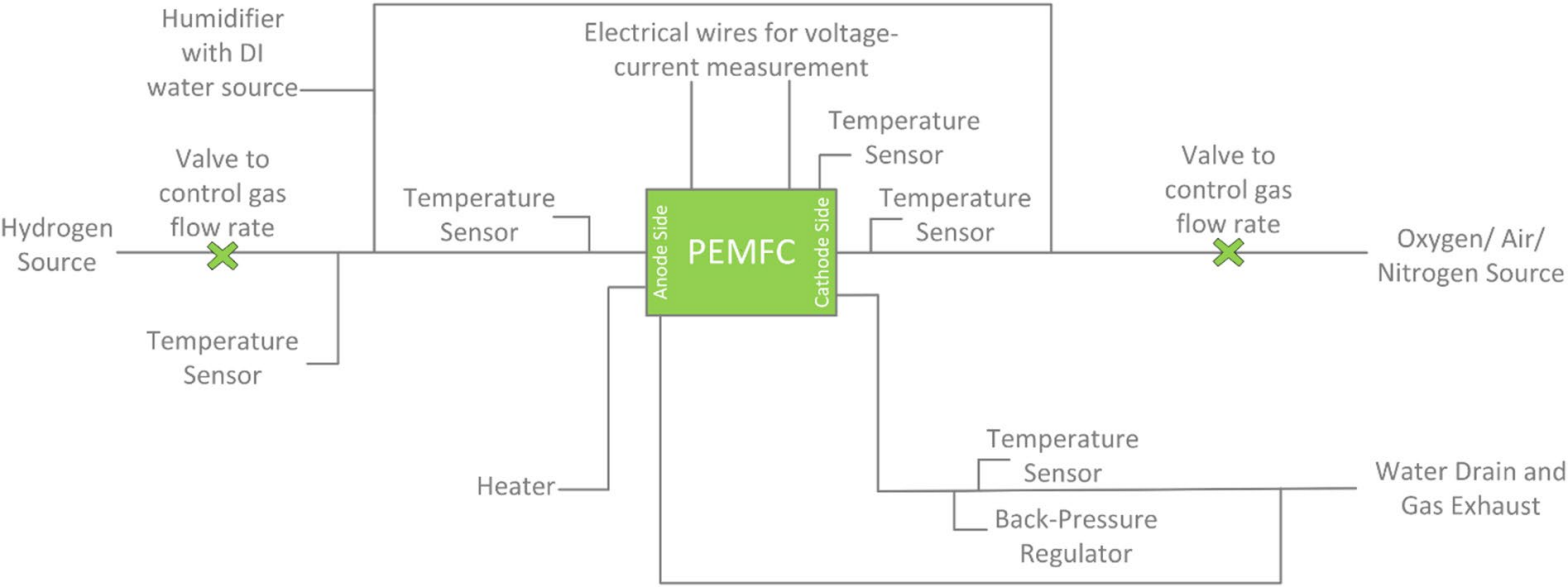
Experiment Setup.

Once the assembly is complete, the fuel cell is subjected to operational testing to evaluate its performance under different conditions, as illustrated in Fig. 2.

### Activation procedure of the fuel cell

The fuel cell must be activated before it starts operating and undergoing tests to reach the maximum performance that the cell can achieve and thus ensure its stability during the later operation. Throughout activation, the cell’s performance gradually improves until it remains constant, at which point the cell is considered fully activated.

Various activation techniques are commonly applied, such as voltage scanning, voltage stepping, application of high current densities, and air starvation. In this study, activation is carried out through two main stages:

- Break-In Procedure

The current increases incrementally in steps of 0.1 A, with a 4-minute interval at each step, starting from 0 A to 1 A.

This process ensures that hydration is uniform within the cell and the catalyst is properly used^31^.

- Air Starvation Technique

After the break-in procedure, an air starvation cycle is applied by changing the current between 0 A for 30 s and 1.25 A for 2 min.

It is called air/cathode starvation because the air flow rate to the cathode electrode when the current is 0 A is set to 0 NLPM.

This technique is used to induce controlled air starvation, which helps improve catalyst activity and assists in the redistribution of water within the cell^32^.

The cell voltage is continuously monitored throughout the activation process to track performance stabilization and ensure consistent operation before commencing full performance testing.

## Results and discussion

In this part of the study, the fabricated fuel cell performance is assessed under different conditions of temperature, relative humidity (RH) levels, and gas flow rates. Two fuel cells of different MEA compositions will be tested.

The V-I polarization curves are used to assess the performance of the cell being tested, as the attention is on the maximum power obtained from the cell and the highest current value reached before the cell voltage collapses due to the high concentration voltage loss. Based on these values, the best configuration of MEA and the best operating conditions of the cell can be decided.

The first fuel cell tested is fabricated following the standard procedures described in the previous section. This cell is evaluated under two different conditions: first using air as the oxidant and then using oxygen, while keeping all other parameters constant.

In the first test, air is supplied to the cathode with the following operating conditions:

- Temperature = 70 °C.
- Hydrogen flow rate (anode) = 0.5 NLPM.
- Air flow rate (cathode) = 0.5 NLPM.
- Relative Humidity (RH) = 100%.

Gas flow rate can be calculated using the following formula:

Flow Rate = Gas Stoichiometry Constant * Current in Amps * Number of Cells in Stack * Stoichiometry Ratio^33^.

**Fig. 3:**
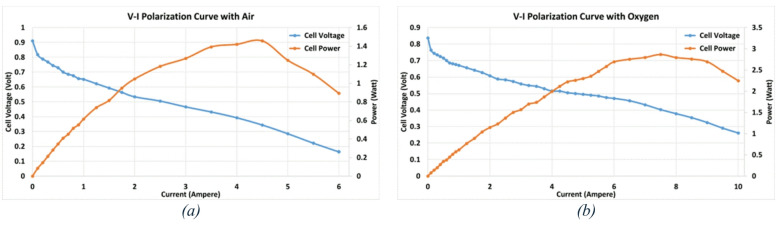
The performance of the tested PEMFC (**a**) using air, (**b**) using oxygen.

Under these conditions, the maximum current achieved is 6 A, with a peak power output of 1.457 W, at 4.5 A and 0.324 V, as shown in Fig. 3 (a).

In the second test, the same cell is operated under the same operating conditions, except that oxygen is supplied to the cathode instead of air. In this case, the maximum current reaches 10 A, with a peak power output of 2.865 W, recorded at 7.5 A and 0.403 V, as shown in Fig. 3 (b).

These results indicate that, compared to air, using pure oxygen as an oxidant greatly improves fuel cell performance. Under the same operating conditions, the higher oxygen concentration enhances the reaction kinetics at the cathode, leading to a higher current density and output power.

The second fuel cell is identical to the first, except that the amount of ink used for spraying the carbon paper during GDL fabrication is doubled. This modification can improve gas diffusion and electrical conductivity by increasing the carbon loading on the GDL and decreasing material loss during spraying. This cell is a loading sensitivity check not a new GDL concept.

This cell is tested under various operating conditions, changing the temperature and relative humidity (RH) at the cathode while maintaining the following parameters constant throughout the test:

- Anode RH = 100%
- Gas flow rate (anode & cathode) = 0.2 NLPM

This cell will make it possible to examine how different operating conditions and higher carbon loading affect fuel cell performance.

**Table 1:** The performance of the tested PEMFC at 100% anode RH, 0.2 NLPM anode and cathode gas flow rate, and different temperatures and RH, using air and oxygen.

		Cathode RH (%)	Max Power (watt)	Voltage at Max Power (Volt)	Current at Max Power (Ampere)	Max Current can be reached (Ampere)
**60** $\rm ^\circ C$	**Air**	50	0.704	0.354	2.044	3
		60	0.721	0.373	1.829	2.5
		70	0.605	0.305	1.854	3
		80	0.718	0.374	1.826	2.5
		90	0.699	0.413	1.708	2.5
		100	**0.745**	0.407	1.982	2.5
	**Oxygen**	50	1.638	0.442	4.087	5.5
		60	1.794	0.413	4.500	6.5
		70	1.272	0.452	2.783	3.5
		80	**1.841**	0.413	4.203	5.5
		90	1.816	0.422	4.323	5.5
		100	1.444	0.500	2.822	3.5
**70** $\rm ^\circ C$	**Air**	50	0.708	0.368	1.846	3
		60	0.796	0.373	2.030	3
		70	0.798	0.344	2.139	3
		80	0.660	0.349	1.861	2.5
		90	0.798	0.427	2.000	2.5
		100	**0.811**	0.432	1.869	3
	**Oxygen**	50	2.198	0.388	5.783	7.5
		60	2.260	0.337	6.486	8.5
		70	2.205	0.392	5.868	7.5
		80	2.088	0.500	4.567	5.5
		90	**2.409**	0.398	6.500	6.5
		100	2.075	0.422	5.172	6.5

**Fig. 4:**
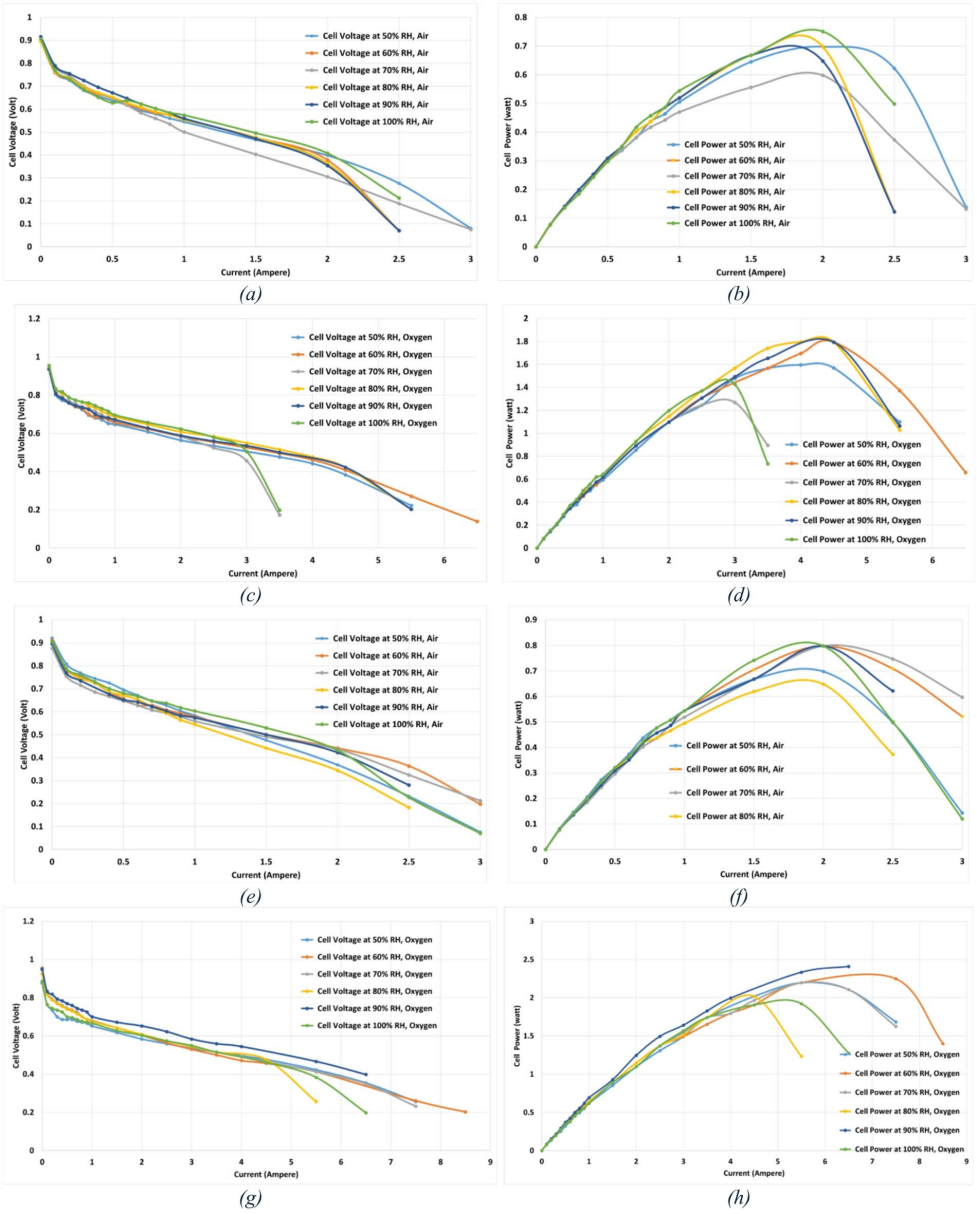
The performance of the tested PEMFC at 100% Anode RH and 0.2 NLPM Anode and Cathode gas flow rate (**a**) Voltage-Current Polarization curve using air at 60 °C and different RH, (**b**) Power-Current curve using air at 60 °C and different RH, (**c**) Voltage-Current Polarization curve using oxygen at 60 °C and different RH, (**d**) Power-Current curve using oxygen at 60 °C and different RH, (**e**) Voltage-Current Polarization curve using air at 70 °C and different RH, (**f**) Power-Current curve using air at 70 °C and different RH, (**g**) Voltage-Current Polarization curve using oxygen at 70 °C and different RH, (**h**) Power-Current curve using oxygen at 70 °C and different RH.

Based on the obtained results shown in Table 1; Fig. 4, it is concluded that:

- Impact of Oxidant gas: When oxygen is used as the oxidant instead of air, fuel cell performance is much improved, and the maximum output power is more than doubled. This is because a higher concentration of oxygen inside the cell improves the reaction kinetics at the cathode.
- Impact of Temperature: The fuel cell performance and maximum output power are enhanced by raising the operating temperature. The maximum output power increases by more than 30% with a 10 °C increase in temperature, demonstrating the relationship between temperature and water management, membrane conductivity, and reaction kinetics.
- Impact of Relative Humidity (RH): Proper membrane hydration increases proton conductivity, improving fuel cell performance at higher RH values. However, two-phase water accumulation at high RH can induce flooding and increase mass-transport losses. Consequently, the RH associated with peak power is governed by a balance between membrane hydration (which lowers ohmic losses) and water accumulation (which raises transport losses), yielding an optimum window rather than a single fixed value. This balance also explains the modest scatter observed among adjacent RH setpoints. So, using air as an oxidant results in the highest maximum output power at range of 90% − 100% RH. And when oxygen is used, it is observed at 80% to 90% RH.

To contextualize performance, the in-lab–fabricated cell is compared with a second cell assembled with a commercial CCM. Using the same structure and GDL, the same assembly procedure and clamping torque, and matched operating conditions, the in-lab cell consistently delivered higher polarization and power metrics. The commercial reference is not tested across the full operating matrix done for the in-lab one; therefore, comparison is reported only at selected conditions where strict parity is available. Representative matched-condition outcomes are summarized in Table 2.

**Table 2:** Comparison between in-lab fabricated PEMFC and commercial CCM PEMFC at 100% anode RH, 90% cathode RH, 0.2 NLPM anode and cathode gas flow rate, and different temperatures, using air and oxygen.

		Max Power (watt) for in-lab fabricated cell	Max Power (watt) for cell with commercial CCM
**60** $\rm ^\circ C$	**Air**	0.699	0.668
	**Oxygen**	1.816	1.417
**70** $\rm ^\circ C$	**Air**	0.798	0.648
	**Oxygen**	2.409	1.921

Direct comparison with previously published PEMFC performance data in the literature is limited in this study due to the multiple modifications made to both the MEA construction and the operating conditions to investigate their combined influence on cell performance. Some of these design parameters and testing conditions are selected based on the best performance reported in the literature with more transparent implementation details, where optimal features from individual studies are integrated into one configuration. Although the comparisons are not fully based on the right scientific sense, the results of this study do provide strong indications of enhanced performance.

## Conclusion

This study is a PEM Fuel Cell lab experiment. It goes through three main phases: the fabrication of the MEA, the assembly of different components of the cell and cell activation, and the cell performance assessment under different operating conditions.

The fabrication phase is to make the GDL and CCM in the lab while ensuring uniform coating distribution, which is a key factor in improving cell performance. Additionally, special attention is given to the quality of the coating process itself as the loss of materials is reduced for cost efficiency as well as to get the loading required for the coating on its substrate. Once the fabrication is completed, the layers are assembled to form the fuel cell, which is then activated and tested.

Two different fuel cells are tested under different operating conditions of temperature, humidity levels, oxidant gas type and gas flow rate. The following are concluded:

- When oxygen is used as the oxidant instead of air, fuel cell performance is much improved, and the maximum output power is more than doubled. This is because a higher concentration of oxygen inside the cell improves the reaction kinetics at the cathode.
- Using another layer of carbon on both sides of the MEA does not significantly affect the cell performance as almost the same results of voltage, electric current and maximum output power are reached.
- Using double the amount of ink during the GDL fabrication in the experiment leads to better cell performance as it increases the carbon loading on the GDL.
- Increased gas flow rate improves cell performance. However, assessing its impact on operational costs is essential, as higher flow rates can lead to increased gas consumption and potential waste of unutilized reactants.
- Increased temperatures during cell operation leads to better performance and higher maximum output power.
- The best value for RH during cell operation is between 80 and 100%. Less than that may lead to membrane dehydration, reducing proton conductivity and lowering cell performance. Above these values, water flooding obstructs the diffusion of gases in the cell and reduces the chemical reaction efficiency.

These conclusions lead to the necessity of balancing the gas flow rate, temperature, and RH during the cell operation to reach the optimal performance, and they also suggest promising directions for low-cost lab-scale fabrication of PEMFCs, with potential applications in academic prototyping and small-scale system integration. The future work of this study is an economic study to reach the best operating conditions at the lowest cost. This will be preparation for transitioning from such in-lab MEA design to pre-commercial applications.

## Data Availability

All data generated or analysed during this study are included in this published article. For any additional clarification, please contact the corresponding author at haya.hesham@eng.asu.edu.eg or hayaheshamalsaid@gmail.com.
